# Molecular Alterations in Sporadic Primary Hyperparathyroidism

**DOI:** 10.4061/2011/275802

**Published:** 2011-09-08

**Authors:** Maria Inês Alvelos, Maria Mendes, Paula Soares

**Affiliations:** ^1^Institute of Molecular Pathology and Immunology of the University of Porto (IPATIMUP), 4200-465 Porto, Portugal; ^2^Faculty of Engineering of the University of Porto (FEUP), 4200-465 Porto, Portugal; ^3^Abel Salazar Biomedical Sciences Institute, University of Porto, 4099-003 Porto, Portugal; ^4^Faculty of Medicine, University of Porto (FMUP), 4200-319 Porto, Portugal; ^5^Cancer Biology Group, IPATIMUP, Rua Dr. Roberto Frias, s/n, 4200-465 Porto, Portugal

## Abstract

Primary hyperparathyroidism (PHPT) is a frequent endocrine disorder characterized by an excessive autonomous production and release of parathyroid hormone (PTH) by the parathyroid glands. This endocrinopathy may result from the development of a benign lesion (adenoma or hyperplasia) or from a carcinoma. Most of the PHPT cases occur sporadically; however, approximately 10% of the patients present a familial form of the disease. The molecular mechanisms underlying the pathogenesis of sporadic PHPT are incompletely understood, even though somatic alterations in *MEN1* gene and CCND1 protein overexpression are frequently observed. The *MEN1* gene is mutated in about 30% of the parathyroid tumours and the protooncogene *CCND1* is implicated in parathyroid neoplasia by rearrangements, leading to an overexpression of CCND1 protein in parathyroid cells. The aim of this work is to briefly update the molecular alterations underlying sporadic primary hyperparathyroidism.

## 1. Introduction

One of the multiple implications of multicellularity is that all parts of a body must be able to communicate with each other, in order to maintain homeostasis. The communication between different parts of an organism is essential for an appropriate response to internal and external environmental stimuli. The endocrine system, through the production, and release of hormones, is a key element for the establishment and maintenance of such regulation [1]. 

The parathyroid glands are the endocrine organs responsible for regulating calcium levels by producing a hormone, the parathyroid hormone (PTH), released directly into the blood [2] that regulates calcium levels acting at various organs.

Calcium plays a fundamental role in controlling the neuromuscular activity, the blood clotting process, bone structure, and integrity of cell signaling. The levels of ionized calcium in bloodstream are maintained by a complex hormonal mechanism, involving three main systems: gastrointestinal system, bone, and kidney [3, 4].

Primary hyperparathyroidism is a common endocrinopathy characterized by an inappropriate oversecretion of PTH. High PTH levels lead to a hypercalcemic state affecting different organs and systems such as bones and kidneys and cardiovascular, gastrointestinal, and nervous systems [5, 6].

The incidence of PHPT is estimated as 25 cases per 100 000 individuals, and the main risk factors are older age, female gender, and exposure to cervical irradiation [7, 8].

This pathological condition, in 80 to 85% of the cases, is due to a parathyroid adenoma (uniglandular disease), followed by 15 to 20% of cases arising due to hyperplasia (multiglandular disease) and rarely, in 2 to 5% of cases, can be caused by a carcinoma [4].

Most of the PHPT cases belong to sporadic forms of the pathology, but about 10% of the cases are familial forms [7]. 

Germline genetic alterations in *MEN1* (*multiple endocrine neoplasia type 1*) gene are associated with PHPT in the context of MEN1 syndrome, and mutations in *HRPT2* (*hyperparathyroidism 2*) gene are associated with HPT-JT (Hyperparathyroidism-Jaw tumor) familial syndrome. The development of PHPT in the context of MEN2 (multiple endocrine neoplasia type 2) syndrome, specifically in the variant MEN2A, is associated with genetic alterations in the *RET* (*rearranged during transfection*) gene. Homozygous or heterozygous mutations in *CaSR* (*Calcium Sensing Receptor*) gene are the source of NSHPT (neonatal severe hyperparathyroidism) and FHH (familial hypercalcemic hypercalciuric), respectively [9, 10].

Although, the genetic alterations associated with familial forms are well known, the genetic alterations underlying sporadic forms are far from being understood. The molecular alterations that are established as being characteristic of sporadic benign parathyroid tumor are genetic alterations in the *MEN1* gene (20 to 30% cases) and Cyclin D1 protein overexpression (30 to 40% of cases) [11, 12]. Parathyroid carcinomas are strongly associated with somatic *HRPT2* mutations [13]. 

### 1.1. The Role of MEN1 Gene in PHPT

The *MEN1* tumor suppressor gene was identified in 1997 as the gene responsible for the autosomal dominant syndrome characterized by tumors of endocrine pancreas, the anterior pituitary and parathyroid glands, the MEN1 syndrome [14]. Apart from being involved in the mentioned familial syndrome, somatic mutations of *MEN1* gene are also implicated in the development of sporadic parathyroid tumors [15]. 

The *MEN1* gene is located on chromosome 11 (band 11q13), consists of 10 exons (with 9 coding exons), and encodes a protein of 610 amino acids called menin that is ubiquitously expressed, at all stages of development [16]. The transcript of *MEN1* gene is a 2.8 Kb mRNA but six more alternative transcripts have been reported, although none of them interferes with the coding region revealing variations of the 5′ translated region only [17]. Despite the high degree of conservation among metazoan, menin does not reveal motifs of known function and has no similarity with any other known protein [18]. 

Tumors of MEN1 patients usually reveal the presence of a germline mutation followed by a somatic alteration such as loss of heterozygosity (LOH) or inactivating mutation, as predicted by the model of Knudson, pointing to *MEN1* gene as a very good example of a classical tumor suppressor gene [19].

About 30% of sporadic parathyroid tumors show *MEN1* gene mutations (see Table 1). These somatic mutations, similarly to what happen with the germline mutations, are spread throughout the coding region. About 40% of these mutations are frameshift, 29% are missense mutations, 18% are nonsense, 7% occur in splice sites, and 6% are deletions or insertions in-frame. Sporadic parathyroid tumors harboring *MEN1* gene somatic mutations frequently evidence LOH on chromosome region 11q13 [20]. Somatic inactivating mutations in this gene have also been identified in other types of tumors, namely, in neuroendocrine tumours such as gastrinomas, insulinomas, lung carcinoids, and pituitary tumors showing a similar loss of function mechanism promoting tumorigenesis [21–23].

From *Drosophila* to humans, menin is conserved but is absent in *C.elegans* and yeast [24]. Subcellular localization studies evidence that the protein has predominantly a nuclear localization and possesses two nuclear localization signals (NLSs) that should be essential for its role in regulation of gene transcription [25]. Menin has been reported to interact with a broad spectrum of proteins involved in regulation of transcription by coordinating chromatin remodeling, genome stability, cell division, and proliferation [26, 27]. 

Recently, it was demonstrated *in vitro* that menin acts as a scaffold and inhibits AKT/PKB activation by regulating its cellular localization, since it hampers the transition from the cytoplasm to the membrane, required to its activation [28]. 

The clinical relevance of pathways and molecular mechanisms in which menin is involved remain to be clarified. 

Most of the *MEN1* gene mutations will lead to a premature stop codon, giving rise to a truncated protein. Frequently this truncated menin may have lost, at least one of its NLS, compromising the protein function, regarding their role of driving the protein to the nucleus, but also by coordinating the regulation of expression of menin target genes [25].

Menin protein can function as suppressor of transcription, because this protein is able to bind a family of transcription factors such as AP-1/Jun-Fos family, and it is also associated with a histone methyltransferase (HMT) complex leading to an increased expression of cyclin-dependent kinase inhibitors (CDKIs) and consequently suppressing cell growth [29, 30]. 

### 1.2. The Role of Cyclin D1 Protein in PHPT

The gene encoding cyclin D1, *CCND1*, located in chromosome 11q13, was initially designated *PRAD1* (*parathyroid adenomatosis 1*) since it was cloned from DNA from a parathyroid adenoma. In 1989, Arnold and colleagues found a genetic rearrangement in a parathyroid adenoma, this rearrangement, inv (11) (p15; q13), positions the 5′ PTH gene regulatory region (located in 11p15) adjacent to the *CCDN1* gene leading to the overexpression of cyclin D1 protein [31]. The increased expression of cyclin D1 promotes the transcription of genes required for DNA synthesis and cell cycle progression. 

Several studies show the overexpression of cyclin D1 in 20–40% of parathyroid tumors (see Table 1), even though they do not demonstrate the presence of the rearrangement, pointing to the presence of other molecular abnormalities leading to an overexpression of this cell cycle regulator [32].

The pathogenic role of this protein was assessed through studies in transgenic mice overexpressing cyclin D1 in parathyroid. These mice were created to model the genetic rearrangement found in human parathyroid tumors [33]. In this model, the overexpression of this cell cycle regulator leads to hyperplasic parathyroid glands, with increased cell proliferation, retaining their capacity of hormone production [34].

### 1.3. Parathyroid Carcinoma and CDC73/HRPT2

In 2002, germline mutations in the tumor suppressor gene *hyperparathyroidism 2* (*HRPT2*) were described by Carpten et al. as being responsible for the HPT-JT familial syndrome [13]. 

*HRPT2* gene is located at 1q25-q31 and encodes a 60 kDa nuclear protein named parafibromin that has been shown to be a member of the polymerase-associated factor (PAF1) complex involved in gene transcription regulation by histone ubiquitination and methylation [35, 36].

Additionally, parafibromin suppresses tumor growth by inducing apoptosis, inhibiting G1 to S phase transition, regulating Wnt canonical pathway, and also regulating gene expression by direct binding of promoter regions, being therefore expected that loss of parafibromin will lead to enhanced cellular proliferation [37–40]. 

The loss of parafibromin as a molecular marker of parathyroid carcinoma was first reported by Tan and colleagues in 2004. These authors noted that loss of parafibromin nuclear staining had a high sensitivity and specificity for the definitive diagnosis of parathyroid carcinoma and their results were confirmed by other groups [41, 42].

Despite its specificity, other studies indicated that, although occurring more frequently in parathyroid carcinomas, loss of parafibromin nuclear staining can also occur in a small proportion of sporadic benign adenomas and therefore cannot be considered exclusive of malignancy. Curiously, these adenomas exhibit cystic features which are commonly observed in the HPT-JT syndrome [43]. Another important aspect is the fact that nuclear positivity cannot exclude the presence of *HRPT2* mutation, once some tumors harboring missense mutations revealed weak nuclear positivity [44]. 

Given the low frequency of parathyroid carcinomas, these are commonly misdiagnosed in the clinical setting and the need of specific markers for this disease is fundamental to reduce potential false-negative cases [45]. Some studies assessed the role of additional molecular marker to complement parafibromin staining in the screening process, emerging the protein 9.5 (PGP9.5) encoded by the *ubiquitin carboxyl-terminal esterase L1* (*UCHL1*), that was found to be upregulated in the majority of parathyroid carcinomas [46]. In contrast, the tumor suppressor *adenomatous polyposis coli* (*APC*) was found downregulated in carcinomas while benign lesions retain the expression [47]. 

In spite of these findings, the unequivocal diagnosis remains a challenge, and additional markers are likely to increase the knowledge and proper recognition of parathyroid carcinomas. 

## 2. The Role of Other Genes in PHPT

The knowledge on the molecular bases of parathyroid tumorigenesis, particularly the sporadic variant, remains largely unknown. Some candidate genes have been studied because of their possible role in sporadic primary hyperparathyroidism development (see Table 1). Despite their involvement in familial forms, no somatic mutations have been found in *RET* and *CaSR* genes [48, 49].

Vitamin D receptor (VDR) has an important role in negatively controlling PTH secretion and parathyroid proliferation, thus representing a good target for this parathyroid pathological condition, but no mutations in either *VDR *gene or vitamin-D-activating enzyme were found in sporadic parathyroid tumors despite the evidence of the reduced expression in some series [62–65].

Abnormal Wnt signaling has been associated with many types of tumors, and deregulation of *CTNNB1* as well as mutations of *LRP5* coreceptor has been found in some series of parathyroid tumors. At variance, other studies pointed out that Wnt/beta-catenin signaling does not seem to contribute to the development of parathyroid tumors [66–68]. 

The existence of common molecular alterations among endocrine tumors and its proximity with *MEN1* gene raised the hypothesis that *SDH5* gene could be involved in parathyroid tumorigenesis, but no genetic alterations were found in this pathology [69]. 

Using techniques such as comparative genomic hybridization (CGH), various authors verified the presence of chromosome gains and losses in specific regions, suggesting the presence of unidentified oncogenes and tumor suppressor genes, which may have a role in parathyroid tumorigenesis. Chromosomal regions in 1p, 6q, 9p, and 13q were found to be lost in benign and malignant parathyroid lesions, indicating these chromosomal regions as eventual carriers of novel tumor suppressor genes. Unknown oncogenes may be present in chromosomal regions of 9q, 16p, and 19p, because several authors demonstrated gain in these loci, in parathyroid tumors [70–72]. 

Chromosomal imbalances have been recognized as a mechanism able to alter the expression of miRNAs. Corbetta and collaborators explored the miR expression profile in parathyroid carcinomas, since the expression of these small noncoding RNAs varies between cancer and normal cells. These authors went to verify differential expression among parathyroid carcinoma and normal tissue. By real-time PCR, it was observed that the overall miRNA expression could separate normal versus cancer tissue and four miRs (miR-296, miR-139, miR-222, and miR-503) revealed differences between tumoural and normal parathyroid tissues [73]. Some genes such as *human growth factor-regulated tyrosine kinase substrate* (*HGS*) and *p27/Kip1* are described as potential targets of these miRNAs but further information about the biological relevance of these findings is needed and might provide tools for identifying new diagnostic and therapeutic strategies [74, 75]. 

Most of the studies in parathyroid tumorigenesis field have been performed using genomics and immunohistochemical approaches [53, 76]. Giusti and collaborators carried out comparative analysis to examine the changes in protein profile between adenomas and normal parathyroid tissue [77]. These authors verified the presence of 30 deregulated proteins in parathyroid adenomas, 22 of them being overexpressed when compared to normal parathyroid tissue [77]. Some of the identified proteins belong to the same functional class of the ones identified by Haven and co-workers when they preformed tissue microarrays for benign and malignant tumors [78]. Overall, these findings represent promising steps for the improvement of the knowledge about this pathology. 

## 3. Concluding Remarks

Presently, alterations in *MEN1* and *CCND1* are still the main genetic alterations associated with the development of sporadic benign tumors (accounting for approximately 30% of the cases). The *HRPT2* gene is not only responsible for the HPT-JT syndrome but also mutated in the majority of parathyroid carcinomas.

A number of other candidate genes (due to their genomic localization, role in familial syndromes, and/or biological function) have been studied in parathyroid tumors, but without promising results. 

Future goals include the identification of additional oncogenes and/or tumor suppressor genes in parathyroid lesions and understanding the molecular insights in the relationship between parathyroid proliferation and hormone regulation. 

## Figures and Tables

**Table 1:** Summary of the molecular alterations associated with familial and sporadic parathyroid tumors.

	*MEN1 *[20, 50–52]	*CCND1* [31, 53–55]	*HRPT2* [13, 56–58]	*RET * [48]	*CaSR* [49, 59–61]
Germline mutations	Inactivating mutations, LOH	—	Inactivating mutations, LOH	Activating mutations	Inactivating mutations
(Familial syndrome)	(MEN1)		(HPT-JT)	(MEN2A)	(NSHPT/FHH)

*Benign*					
Somatic mutations	Inactivating mutations, LOH	Activating Inv (11) (p15; q13)	Inactivating mutations	N	N
Prevalence of somatic alteration	20 to 30%	~5%	2 to 4%	—	—
Protein expression (%)	Downregulation (20 to 40%)	Overexpression (30 to 40%)	Downregulation (ND)	ND	Downregulation (Up to 90%)

*Malignant*					
Somatic mutations	Inactivating	ND	Inactivating mutations, LOH	N	ND
Prevalence of somatic alteration	~13%	ND	70 to 100%	—	—
Protein expression (%)	ND	Overexpression (~90%)	Downregulation/loss of expression (70 to 100%)	ND	Downregulation (~30%)

N: negative; ND: not determined.
